# Influence of encapsulation on the survival of probiotics in food matrix under simulated stress conditions

**DOI:** 10.1016/j.sjbs.2022.103394

**Published:** 2022-07-25

**Authors:** Muhammad Afzaal, Farhan Saeed, Muzzamal Hussain, Zoria Ismail, Azhari Siddeeg, Ammar AL-Farga, Moneera O. Aljobair

**Affiliations:** a Department of Food Sciences, Government College University Faisalabad, Pakistan; b Department of Food Engineering and Technology, Faculty of Engineering and Technology, University of Gezira, Wad Medani, Sudan; c Department of Biochemistry, College of Sciences, University of Jeddah, Jeddah, Saudi Arabia; d Department of Physical Sport Science, College of Education, Princess Nourah bint Abdulrahman University, P.O. Box 84428, Riyadh 11671, Saudi Arabia

**Keywords:** Probiotic, Viability, Encapsulation, Hydrogels, Simulated digestion conditions, WPI, Whey protein isolate, SA, Sodium alginate, AOAC, Association of Official Analytical Chemists, SGJ, Simulated gastric juice

## Abstract

The main objective of the present study was to evaluate the influence of encapsulation by extrusion technique using two hydrogels, namely; sodium alginate (Na-ALG) and whey protein isolate (WPI) on *Bifidobacterium bifidium* viability and stability of yoghurt under simulated gastrointestinal conditions. Probiotic bacteria (free or encapsulated) were added to yogurt for four weeks to test their viability and stability. Physicochemical and sensory analysis of yoghurt were conducted. Viability of *B. bifidium* in the simulated gastrointestinal conditions pH 2 and pH 7.5 was determined. Also, the efficiency of encapsulated final yield of the microcapsules was determined. With storage time, the pH of yoghurt containing encapsulated bacteria increased more than that of yoghurt containing free probiotic bacteria, resulting in a decrease in acidity. When compared to yoghurt containing encapsulated bacteria, the lactose level of yoghurt containing free probiotic bacteria decreased over time. The viscosity of yoghurt containing encapsulated WPI remained stable over the storage period, with syneresis remaining stable. The sensory properties of yoghurt containing free probiotics deteriorated over time. Cell viability was significantly reduced in yoghurt-containing free probiotics compared to other treated yoghurts. Cell viability in free probiotics yoghurt was lower than in encapsulated ones when exposed to simulated gastric and intestinal juice. In conclusion, WPI- encapsulated probiotics showed better stability over 28 days of storage in both yoghurt and gastrointestinal conditions, followed by sodium alginate.

## 1 Introduction

Probiotics are defined by the World Health Organization as “living microorganisms which upon ingestion in certain numbers, exert health benefits beyond inherent general nutrition” (FAO/WHO, 2020). Hill et al. (2014) reported that probiotics are described as “live beneficial micro-organisms that, when ingested in sufficient quantities, boost up host's immunity against intestinal pathogens and prevent an array of gastrointestinal disorders”. Probiotic bacteria are the constituents that mostly fermented the food, enhancing its digestibility and therapeutic potential (Lourens-Hattingh and Viljoen, 2001). According to Ramos et al. (2018), some probiotic bacteria, such as *Lactobacillus johnsonii*, L. *rhamnosus*, and *Saccharomyces boulardii*, provide a healthy gut flora and contribute to the host’s health. Several gut microbiomes research is rapidly rolling because of the accessibility of consistent tools and novel analysis of microbes (Guarner, 2014).

Probiotic health benefits include improving lactose intolerance symptoms, lowering cholesterol, anti-cancer property, antibiotic therapy, and reducing diarrhea incidence (Oak and Jha, 2019). The probiotics viability and survival are naturally low in yoghurt, and the recommended level is approximately 10^8^ – 10^9^ cells in the product (Afzaal et al., 2019). The probiotics viability in fermented food is influenced by various factors, which include extrinsic and native features such as the production of hydrogen peroxide, post acidification, oxygen, pH, storage temperature, and processing conditions (Shah, 2000). Products of dairy have the capability of freeze injury, reduce oxygen toxicity, and constancy of probiotics (Fenster et al., 2019). The survival of probiotics in the GIT conditions is vital for promoting the health benefits (Kechagia et al., 2013).

Encapsulation is a technique to provide physical protection to the bioactive elements besides the chemical degradation and maintaining their efficiency, especially for the food industry. It is an evolving technique that allows the maintenance of microbial isolates (Đorđević et al., 2015). Microencapsulation is a powerful technique commonly used for the protection of a wide range of biomolecules such as small molecules and protein and cells of bacterial, yeast, and animal. (Borgogna et al., 2010). The safe release of probiotic bacteria in the human gut is a major issue after storage. In the human gut, several microbes play a significant role in human health by improving the absorption of numerous metabolites through the gastrointestinal tract (Mahammadi et al., 2013).

The most economical and simplest method is an extrusion that uses a mild operation that gives great probiotic viability with minimal damage to the probiotic cells (Krasaekoopt et al., 2003). Concentrations of alginate and calcium chloride that are mostly used are 1–2 % and 0.05–1.5 M, respectively. Size varies in diameter from 2 to 3 mm (Krasaekoopt et al., 2003). Whey protein isolate is a new approach as encapsulating material that is helpful in targeted delivery and alternative to medicinal health care (Shi and Lee, 2020). Milk proteins act as an encapsulating material and can form a gel in this situation just like gelatine. Probiotic cells have milk protein which acts as a natural vehicle and utilizes as a release system in their physicochemical along with structural properties (Livney, 2010). Encapsulation of bacteria is expected to extend cells viability and retain the quality attributes of yoghurt. The primary goal of this study was to determine the effect of encapsulation by extrusion using two hydrogels, sodium alginate (Na-ALG) and whey protein isolate (WPI), on the viability and stability of *Bifidobacterium bifidium* in yoghurt under simulated gastrointestinal conditions.

## 2 Materials and Methods

### 2.1 Materials

The culture of the probiotic (*B. bifidum*) *B. bifidum* PRL2010 or *B. bifidum* NCIMB 41,171 or *B. bifidum CNCM I-4319 or B. bifidum BGN4* was provided by NIFSAT, University of Agriculture Faisalabad. The chemicals and reagents were procured from a local market and scientific store. The present study was conducted at the Food Safety and Biotechnology lab, Government College University Faisalabad, Pakistan. All chemicals and reagents used are of reagent grade.

### 2.2 Activation of microbial strain

The probiotic culture was activated by following the process of Fareez et al. (2015) with some modifications. *B. bifidum* was anaerobically grown using de Man–Rogosa–Sharpe (MRS) broth (BD Difco™, Franklin Lakes, NJ, USA) with supplementation of 0.05 % (w/w) l-cysteine-HCl (Sigma, St. Louis, MO, USA) at 37C for 28 h. To get a high bacterial population, the strain was sub-cultured three times, afterwards, the cell pellets were harvested at 4000 rpm by centrifugation for 20 min. The pellets were washed using sterile NaCl solution (0.9 % (w/v) and recovered by centrifugation at 4000 rpm for 20 min. After that, the cells were dissolved in sterile NaCl solution (0.9 % (w/v). Finally, the suspension was used immediately for further treatments.

### 2.3 Micro-encapsulation

Microencapsulation beads were prepared according to the process described by Ayama et al. (2014) with little modification. The probiotic culture of *B. bifidum* was taken and encapsulated with a 1.71 % % sodium alginate (SA) solution using the extrusion method. Both of the solutions were mixed with a ratio of 1:1. To stabilize the microencapsulated beads, the emulsion was lowered into the sterile solution of CaCl_2_ (0.l M). Then beads were attained. In another experiment whey protein (6 %) was prepared according to Giroux and Britten (2011) method and was used as an encapsulating material. The probiotic solution was mixed with the solution of WPI of 100 ml for 30 min with moderate shaking on a magnetic stirrer. Beads encapsulated with WPI were attained, washed, collected, and stored for future use.

### 2.4 Characterization of microbeads

#### 2.4.1 Size

To assess the prepared microcapsules, Abdelbary et al. (2012) standardized method of optical microscopy was used.

#### 2.4.2 Encapsulation efficiency

The efficiency of encapsulated microbeads or the final yield of the microcapsules was determined as the process explained by Zou *et al*., (2011).

Encapsulationyield=N/No×100

### 2.5 Yoghurt fermentation

Yogurt preparation was done by using the procedure of Mousa et al. (2014). Milk was pasteurized at a temperature of 60 °C for 30 min. Also, standardized to 3.5 % fat. Then cooled it at 40–43 °C. The culture was prepared and inoculated with 100 mg starter culture in 50 ml of milk. The milk was poured into the sterilized cups and fermented at 42-40 °C for 5–6 h. The details are given in Table 1. *B. bifidum* was added to milk with or without encapsulation. Yoghurt was analyzed for 28 days of storage. The treatment plan was as follows: To, control sample; T1, free probiotics; T2, encapsulated with alginate; T3, encapsulated with whey protein isolate.

### 2.6 Physicochemical analysis of yoghurt

#### 2.6.1 pH

The pH of the product was determined by using the procedure given by AOAC (2006). pH meter, which was formerly calibrated with typical solutions of buffers of pH 9, 4, and 7.

#### 2.6.2 Lactose

The method of AOAC (2000) was applied to determine lactose content in yoghurt.

#### 2.6.3 Acidity

The acidity determination was carried out by the titration method described by AOAC (2006). The procedure was followed by taking 20 ml distilled water into the sample of 100 g, and indicator 2 ml phenolphthalein was added up. Then a standard solution of 0.1 mol/L sodium hydroxide was dropped into the sample solution awaiting a change of color. Acidity was determined following the equation below:

Acidity=c×v×100/m×0.1

C is the concentration standard, V is the volume in ml, and m (g) is mass.

#### 2.6.4 Viscosity

A viscometer (Brookfield LVAVE‐2130) was used to determine viscosity as described by Afzaal et al. (2019) method.

#### 2.6.5 Syneresis

Syneresis was measured by following the procedure of Ayar and Gurlin (2014).

### 2.7 Yoghurt sensory evaluation

The sensory of all types of yoghurt samples was carried out using 9-hedonic scale as previously reported by Farinde et al. (2009).

### 2.8 Microbiological analysis

#### 2.8.1 *B. Bifidium* viability in yoghurt

The viable count of yoghurt was checked by Shi et al. (2000). The dilutions were prepared in saline solution. The samples were placed on MRS agar for 48–72 h at 37 °C.

#### 2.8.2 Free and encapsulated *B. Bifidium* viability in simulated gastric conditions (*in-vitro*)

The viability of *B. bifidum,* either in free or encapsulated form was subjected to a simulated gastric fluid having pH 2. The viability was determined by the method described by Bosnea et al. (2014).

#### 2.8.3 Viability of *B. Bifidium* in simulated intestinal fluid (in-vitro)

Free and encapsulated probiotics viability was estimated by subjecting the probiotics to SIF having a pH of 7.5. The viable cells were counted, as reported by Bosnea et al. (2014).

#### 2.8.4 Statistical analysis

Each sample was thoroughly examined three times. SPSS statistical software was used to analyze the data (version 25, IBM Corp., Melbourne, Australia). The obtained data was presented as the mean ± standard deviation. To determine differences between means, a one-way analysis of variance (ANOVA) was used.

## 3 Results

### 3.1 Characterization of microcapsules

#### 3.1.1 Morphological analysis

The morphology of resulted microcapsules (sodium alginate (SA) and whey protein isolate (WPI) beads) was normal and circular in shape, the color showed opaque white and the size ranged from 1.53 to 1.90 µm. While for others, no obvious difference was observed, the size differed from 1.33 to 1.57 µm. Beads of *B. bifidium* had shown reliability within the capsule. The existence of cells confirmed the functionality of the microencapsulation technique*.*

#### 3.1.2 Encapsulation efficiency

Table 1 shows the probiotics' cell release when encapsulated with sodium alginate (SA) or whey protein isolate (WPI). The efficiency of encapsulation was higher with sodium alginate. Encapsulation has improved the persistent presence and stability of the probiotic cell in the product and its protection during processing and other environmental conditions.

**Table 1:** Encapsulation efficiency.

Treatments	Numbers before encapsulation	Numbers after encapsulations	Efficiency %
T_2_	8.64	8.52	99 %
T_3_	8.52	8.21	95 %

There are no significant differnces at P ≤ 0.05 between all treatment in pH at the 0th day, but T_o_ was lowered significantly in from the 7th day to the 28th day in compare with the other treatments, while T1 was lowered significantly in from the 14th day to the 28th day. And no significant differences were appeared in pH between both T2 and T3 at all time.

### 3.2 Physicochemical analysis of yoghurt

#### 3.2.1 pH and acidity

**Fig. 1:**
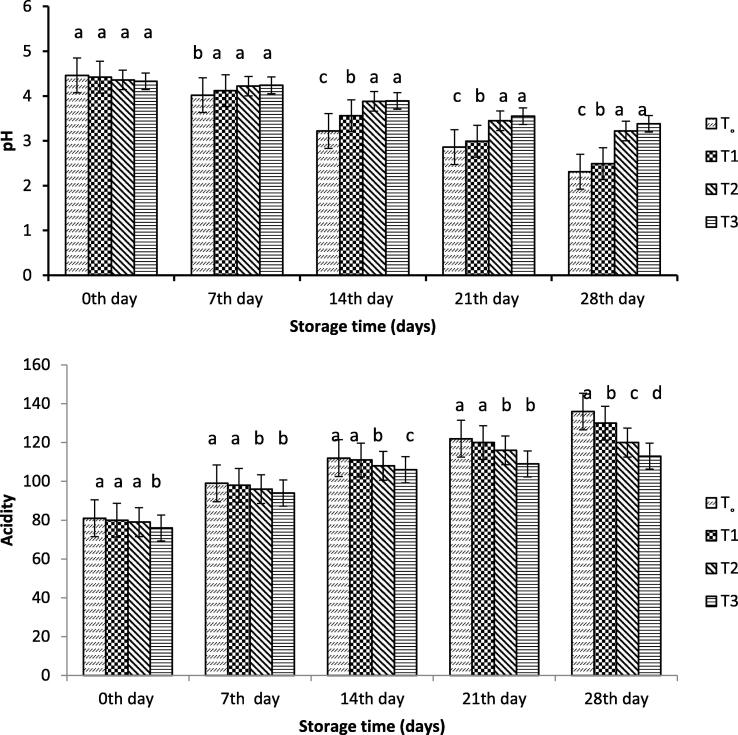
Effect of free and encapsulated probiotics on pH (top) and acidity (bottom) of yogurt. T_o_ = control yogurt T1 = yogurt with free probiotic bacteria T2 = yogurt containing probiotic bacteria with NA-ALG encapsulation T3 = yogurt containing probiotic bacteria with WPI encapsulation.

To assess the stability of the encapsulated probiotic bacteria during storage, the values of pH and acidity were measured as shown in Fig. 1. The yoghurt with and without free probiotic bacteria showed a significant decrease in the pH value during the storage days, and the pH value significantly decreased. Yoghurt with and without free probiotic bacteria initial pH was 4.46 and significantly dropped to 2.31and 2.61 with time, respectively. However, the rate of reduction in pH of yoghurt containing SA-encapsulated probiotic bacteria or WP isolate was low. At the end of the storage period, yoghurt encapsulated with WP isolate showed higher pH than the free probiotic bacteria and probiotic bacteria encapsulated with ALG. In addition to the pH, the addition of microcapsules had a significant effect on the acidity of yoghurt. In yogurt-treated encapsulated probiotic bacteria, there was an increasing trend in acidity but lower than that of yoghurt with and without free probiotic bacteria.

#### 3.2.2 Lactose

**Fig. 2:**
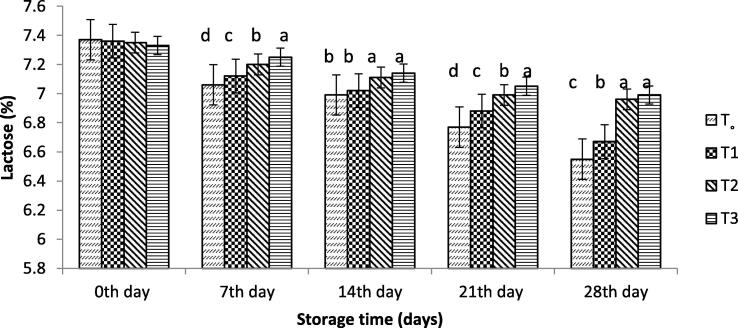
Effect of free and encapsulated probiotics on lactose (%) of yogurt. T_o_ = control yogurt T1 = yogurt with free probiotic bacteria T2 = yogurt containing probiotic bacteria with NA-ALG encapsulation T3 = yogurt containing probiotic bacteria with WPI encapsulation.

Fig. 2 shows the lactose content of different yoghurt samples. Lactose concentration was significantly decreased with storage time. The value of lactose during storage days decreased significantly in yoghurt with and without free probiotic bacteria compared to that containing SA-encapsulated probiotic bacteria or WP isolate. A significant decrease in lactose was observed in yoghurt with and without free probiotic bacteria, which was found to be 6.67 % and 6.52 %, respectively, at the end of the storage period. Yoghurt treated with bacteria encapsulated with alginate and that encapsulated with WPI showed less fluctuation in lactose content during storage compared to yoghurt containing free probiotic bacteria.

#### 3.2.3 Viscosity and syneresis

**Fig. 3:**
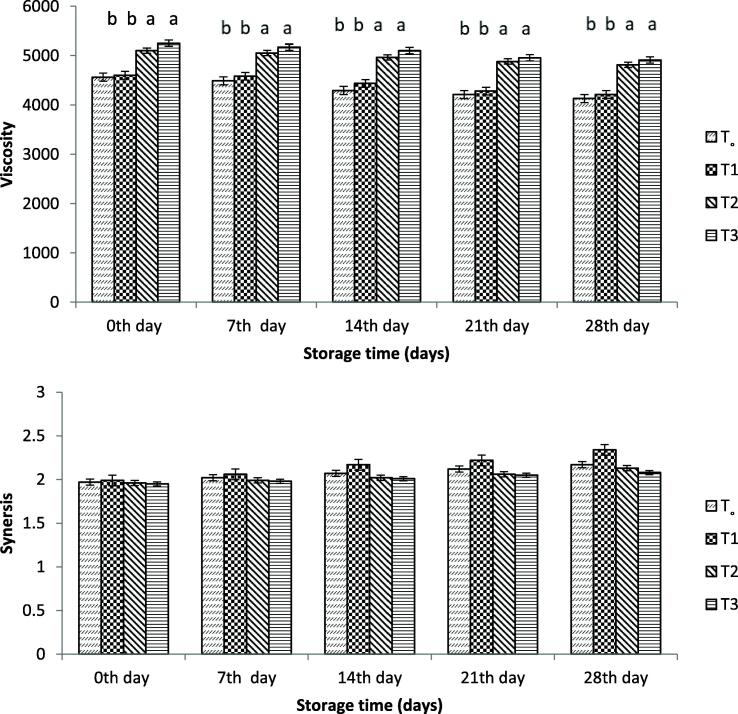
Effect of free and encapsulated probiotics on (top) viscosity (cp) and (bottom) syneresis (ml) of yogurt. T_o_ = control yogurt T1 = yogurt with free probiotic bacteria T2 = yogurt containing probiotic bacteria with NA-ALG encapsulation T3 = yogurt containing probiotic bacteria with WPI encapsulation.

Fig. 3 summarizes the impact of different treatments and storage time on viscosity and syneresis. The viscosity of yoghurt was slightly decreased with time. The viscosity of the yoghurt with and without free probiotic bacteria was significantly reduced at the end of storage period. Less variability in yogurt containing probiotic bacteria with WPI encapsulation with a maximum value of 5.25 cP and a minimum value of 4.91 cP was observed during storage. At the end of the storage period, yoghurt with and without free probiotic bacteria showed a viscosity of 421 and 430 cp, respectively.

Syneresis of yoghurt with different treatments during storage is shown in Fig. 3**.** The value of syneresis was slightly increased with the storage period for all treated yoghurts. The maximum value was observed on the 28th day of storage. Yoghurt containing no probiotic bacteria showed a minimum value of 1.97 ml on the first day and gradually increased to 2.17 ml with time while that containing free probiotic bacteria showed a maximum syneresis of 2.34 ml. The increase in syneresis level could be due to an increase in acid production as well as proteolytic activity. A minimum value was observed in containing WP-encapsulated probiotic bacteria (1.95 ml) on the first day and increased to 2.08 ml on the 28th day of storage. The encapsulated bacteria showed a slower production of lactic acid compared to the control samples (T_o_), which showed 2.17 ml of synergism on day 28, and that was lower than the yoghurt produced with free *Bifidobacterium bifidium*.

### 3.3 Sensory evaluation

**Fig. 4:**
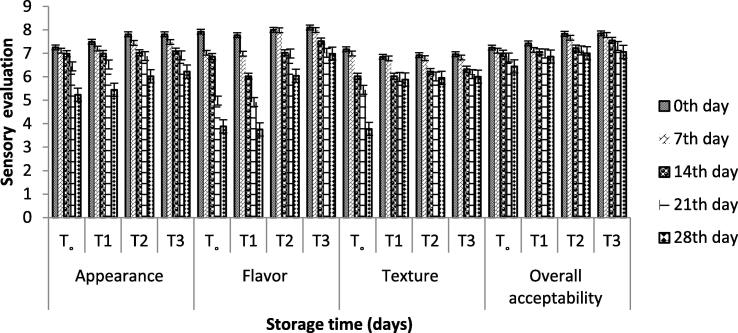
Effect of free and encapsulated probiotics on sensory characteristics of yogurt. T_o_ = control yogurt T1 = yogurt with free probiotic bacteria T2 = yogurt containing probiotic bacteria with NA-ALG encapsulation T3 = yogurt containing probiotic bacteria with WPI encapsulation.

The average values of all treatments for appearance, flavor, texture, and overall acceptability were decreased during storage for 28 days, but the score of sensory evaluation within the treatments increased with due respect as shown in Fig. 4. When probiotic bacteria were encapsulated, it limited the production of acid, increasing the product's acceptability. Encapsulated probiotic bacteria did not affect the taste, flavor, texture, and overall acceptability of the product, but the customer felt the grainy texture. The flavoring parameter lessened mainly due to the degradation of fragrant compounds present in the product. The flavor was affected by a slight increase in its sharpness which was produced by the change in microbial value.

### 3.4 Microbiological analysis

#### 3.4.1 *B. Bifidium* viability in yoghurt

**Fig. 5:**
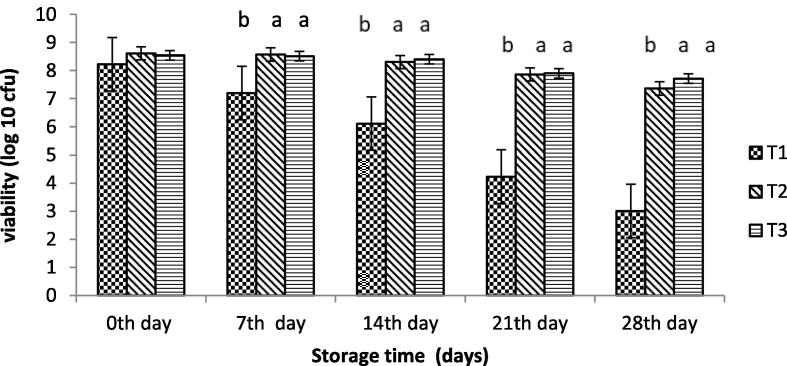
Effect of encapsulation on the viability of probiotics in yogurt (cfu/ml). T_o_ = control yogurt T1 = yogurt with free probiotic bacteria T2 = yogurt containing probiotic bacteria with NA-ALG encapsulation T3 = yogurt containing probiotic bacteria with WPI encapsulation.

A vital prerequisite of cultures of probiotics to be used as dietary addition is that the microorganisms should sustain their viability upon storage (Ding & Shah, 2008). The original cell count of encapsulated probiotics bacteria with SA and WPI was 8.50 and 8.63 log cfu/ml in yoghurt, as shown in Fig. 5. Probiotic bacteria encapsulated with whey protein isolate showed better results than other treatments at 4 °C. The viability of probiotics bacteria encapsulated with SA reduced from 8.61 log cfu/ml to 7.36 log cfu/ml while that with WPI showed a maximum value of 8.54 log cfu/ml and a minimum value of 7.71 log cfu/mL. However, yogurt with free probiotic bacteria showed a significant decrease in viable cells with storage time.

#### 3.4.2 Free and encapsulated *B. Bifidium* viability in simulated gastric conditions and simulated intestinal conditions (*in-vitro*)

**Fig. 6:**
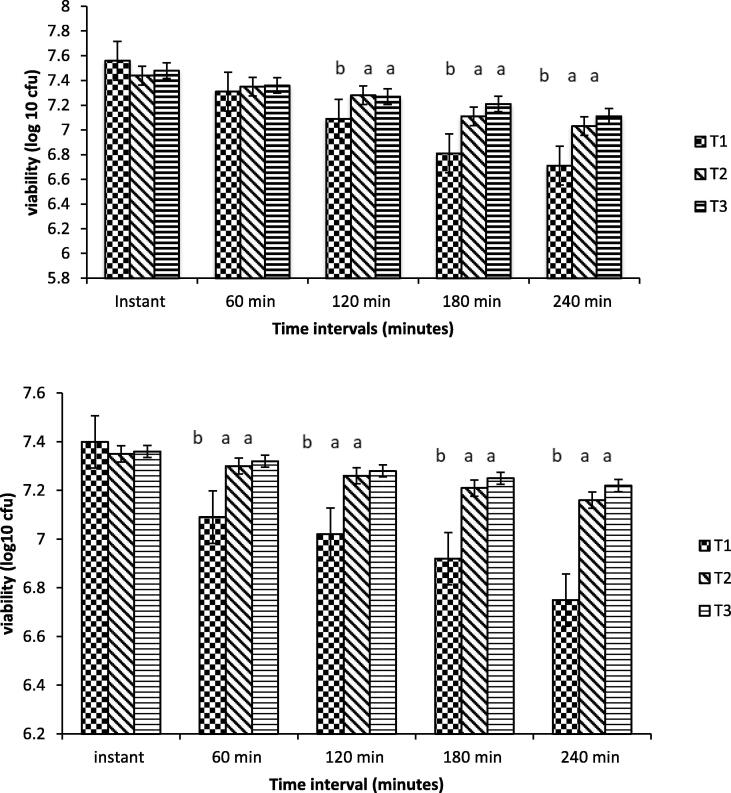
Viability of free and encapsulated probiotics under simulated (top) gastric juice and (bottom) intestinal juice (cfu/ml). T1 = free probiotic bacteria T2 = Encapsulated probiotic bacteria with NA-ALG T3 = Encapsulated probiotic bacteria with WP.

The viability of *B. bifidium* in free or encapsulated form with sodium alginate and whey protein isolate in MRS broth containing 0.3 % pepsin and pH 2 is illustrated in Fig. 6. The number of probiotic bacterial cells was affected by storage time. The product showed a better result when *B. bifidium* was encapsulated with whey protein isolate. As the count difference of 0.37 log cfu/ml was observed. The viability was enhanced when probiotics were covered with whey protein isolate, while all other treatments showed more variation in count rates. An in-vitro study showed the viability of probiotics in yoghurt, as shown in Fig. 6. The number of free cells of *B. bifidium* was reduced significantly. The most damaging effect was observed for unencapsulated probiotics, as indicated by a significant decrease in log cfu/g. While the product containing B. bifidium encapsulated with sodium alginate was slightly reduced, the product containing B. bifidium encapsulated with whey protein showed a very slight decrease in log cfu/g. As a result, when compared to free probiotics cells and encapsulated with SA, B. bifidium encapsulated with WPI provided better protection.

## 4 Discussion

### 4.1 Morphology and efficiency of encapsulation

In terms of morphology and encapsulation efficiency, the size of microcapsules obtained in this study is lower than that reported by Arepally et al. (2022), who discovered that the size of microcapsules ranged from 82 to 149.37 µm. Generally, in the fermentation process, the microcapsule size is less than 1 mm, which is the source of mechanical instability. The stability of probiotics has been extended in fermented food applications, with a volume of 1 to 3 mm is the most valuable. When microcapsules were oversized above standard, they showed a negative sensorimotor effect (Heidebach et al., 2012). The efficiency of encapsulation demonstrates the effective release of cells through the cap to a specific site. Survival depends on two factors, i.e., the type of probiotics and the encapsulating material (Cook et al., 2012).

### 4.2 Physicochemical analysis of yoghurt

Compared to yoghurt containing probiotic bacteria encapsulated with SA or WPI, a significant decrease in the pH value of the yoghurt with and without free probiotic bacteria was observed during the storage days. This result was consistent with those reported by Kailasapathy (2006), who concluded that yoghurt pH value was high with the probiotics bacteria encapsulated with alginate-starch compared to yoghurt containing free probiotic bacteria. The resultant pH decrease was driven by acidity value with the change of lactose to lactic acid during the storage period, as reported by Kailasapathy (2006). Accordingly, yoghurt treated with bacteria encapsulated with alginate and that with WPI showed less fluctuation in lactose content. This difference is due to the lower consumption of lactose in the encapsulated probiotic bacteria that use less lactose, thus, slow variations in acidity and pH are observed. Yogurts treated with encapsulated ALG and WPI showed lower variability in viscosity values, with the encapsulation materials carrying the ability to stabilize. Shihata and Shah (2002) reported that the starter bacteria of diverse types consequently showed a change in viscosities of yoghurt during storage time. The starter bacteria contained enzyme proteases (Toledano et al., 2011) which acted on the yoghurt protein matrix and ultimately lowered the value of viscosity. Patocka et al. (2006) reported that the addition of soluble whey protein to yoghurt reduces the viscosity of the product. The encapsulated bacteria produced lactic acid at a slow rate compared to the control samples, which showed slower synergism than the yoghurt produced with free *Bifidobacterium bifidium*. Aryana et al. (2006) reported that the whey separation of yoghurt occurred and showed a rapid separation during the first week, which is similar to the result of the present study.

### 4.3 Sensory evaluation

Sensory attributes of the yoghurt produced with encapsulated probiotic bacteria were not significantly affected even during storage. The flavouring parameter lessened mainly due to the degradation of fragrant compounds present in the product. The flavour was affected by a slight increase in its sharpness which was produced by the change in microbial value. Similar results were reported by Ott et al. (2000). The flavour depends on compounds fragrant and adds firmness to the end product (García-Ceja et al., 2015).

### 4.4 Microbiological analysis

Compared to yoghurt containing SA- or WP-encapsulated probiotics or that exposed to simulated gastric juice (SGJ) or intestinal juice, yoghurt with and without free probiotic bacteria showed less survival rate with storage time. The results obtained in this study agree with that of Sohail et al. (2011), who reported that the probiotic bacteria encapsulated with alginate beads is of great importance for improving survival in harsh acidic and yellow environments and also in food matrices. Moreover, a study showed that the low pH improved the viability to some extent as well as encapsulation with the culture of planktonic, which affected the viability (Gbassi et al., 2009). Different strains of probiotic bacteria showed different responses when they came in contact with acid and bile (Bosnea et al., 2014). The present research showed that the whey protein isolates were more efficient in their defensive action due to the composition of amino acids that offered a defensive environment for strains of probiotics cells. Several studies have found that there are significant differences in probiotic strain survival in acidic environments. The probiotic culture's sensitivity to acidity is exacerbated by the fact that acidity can increase during storage, a phenomenon known as “over-acidification.” According to Kailasapathy (2006), this post-acidification during storage is caused by β-galactosidase, which is still active at 0–5 °C. In this case, the pH may fall below 4.2, resulting in whey separation and a decrease in viability due to hydrogen ions rather than lactate ions. Yoghurt having probiotics bacteria encapsulated with whey protein isolate showed effective results when the product came into contact with simulated gastric juice (SGJ). It was observed that microencapsulation is acted as a shielding material for probiotic microorganisms against adverse environmental conditions. Interestingly, the components of the yogurt seemed to provide an improved shelter for the cells of the probiotic (*B. bifidium*). Anyhow, WPI encapsulation showed an extra effective response when exposed to SGJ compared to free and encapsulated with SA.

## 5 Conclusion

According to the findings of this study, microencapsulation significantly preserves the quality attributes of yoghurt and increases the survivability of *Bifidobacterium bifidium* in yoghurt. Whey protein isolates encapsulated in probiotics outperformed SA-encapsulated probiotic bacteria with and without free probiotics. Encapsulation showed a slight decrease in viable count over the course of the product's storage time. Dairy foods are the most effective means of delivering probiotic bacteria to the human GI tract. As a result, the probiotic cells must first encapsulate before being added to dairy foods.

## Declaration of Competing Interest

The authors declare that they have no known competing financial interests or personal relationships that could have appeared to influence the work reported in this paper.
